# Palmitoylation Code and Endosomal Sorting Regulate ABHD17A Plasma Membrane Targeting and Activity

**DOI:** 10.3390/ijms262010190

**Published:** 2025-10-20

**Authors:** Byeol-I Kim, Jun-Hee Yeon, Byung-Chang Suh

**Affiliations:** Department of Brain Sciences, Daegu Gyeongbuk Institute of Science and Technology (DGIST), Daegu 42988, Republic of Korea; starkim@dgist.ac.kr (B.-I.K.); jhyeon@dgist.ac.kr (J.-H.Y.)

**Keywords:** ABHD17, palmitoylation, depalmitoylase, lipid modification

## Abstract

Protein S-palmitoylation is a reversible lipid modification that regulates various aspects of protein function, including membrane association, subcellular localization, trafficking, stability, and activity. The depalmitoylase ABHD17A removes palmitate from multiple substrates, but its cellular positioning and the role of its own palmitoylation in regulating its function remain unclear. This study identifies a palmitoylation code within the conserved N-terminal cysteine cluster of ABHD17A, which governs its intracellular distribution and plasma membrane (PM) targeting. N-terminal palmitoylation is essential for PM localization. Through the use of code-restricted mutants, we found that modifications in the middle region (C14, C15) are critical for PM targeting and catalytic activity, while modifications at the front (C10, C11) and rear (C18) influence endosomal routing and delivery to the PM. Alanine scanning revealed that adjacent hydrophobic residues, particularly L9 and F13, are crucial for initial engagement with endomembranes. Sequence analysis and mutagenesis identified two tyrosine-based YXXØ motifs within the alpha/beta hydrolase fold; disruption of the proximal motif (L115A) decreased surface abundance and redirected ABHD17A to autophagosomes, indicating a need for YXXØ-dependent endosomal sorting, likely at the trans-Golgi network. Biochemical assays demonstrated a continuum of acylation states influenced by the palmitoylation code. This requirement for the middle region was conserved in ABHD17B and ABHD17C. Overall, our findings suggest a stepwise mechanism for ABHD17A delivery to the PM, enabling its depalmitoylase activity on membrane-bound substrates.

## 1. Introduction

Protein S-palmitoylation is a reversible lipid modification in which long-chain fatty acids are covalently attached to cysteine residues through thioester linkages [1,2]. This modification enhances protein hydrophobicity, promotes membrane association, and modulates subcellular localization, trafficking, stability, and activity, thereby facilitating a wide range of intracellular functions [3,4,5]. Estimates indicate that over 10% of the human proteome is subject to palmitoylation [6,7], and dysregulation of this modification has been implicated various conditions, including cancer [8,9], cardiac arrhythmia [10], melanoma [11], neurodegenerative and neuropsychiatric disorders [12,13,14]. The palmitoylation cycle is catalyzed by zDHHC palmitoyl-acyltransferases (zDHHC-PATs) and reversed by depalmitoylating enzymes, including cytosolic acyl protein thioesterase (APT1/2), the ABHD17 family, and lysosomal palmitoyl protein thioesterase 1 (PPT1) [15,16].

Multiple studies have demonstrated that multisite palmitoylation can encode distinct intracellular fates and functions for a single protein, effectively forming a “palmitoylation code” [17,18,19,20]. For instance, differential palmitoylation patterns dictate the targeting of synaptosome-associated protein 25 (SNAP25) within the secretory pathway [17], and a specific palmitoylation code regulates the assembly of the phosphatidylinositol 4-kinase IIIα (PI4KIIIα) complex, thereby maintaining PI(4,5)P_2_ homeostasis at the plasma membrane (PM) [18]. Additionally, the electrostatic landscape of the Golgi, particularly the phosphatidyl 4-phosphate-dependent negative surface charge, plays a crucial role in recruiting and positioning substrates for palmitoylation [19]. On the depalmitoylation side, APT2 can deform membranes to extract acyl chains from substrates, highlighting the active role of thioesterases in shaping palmitoylation cycles [20].

The ABHD17 family (ABHD17A/B/C) consists of membrane-associated serine hydrolases that depalmitoylate substrates such as Ras isoforms and PSD-95, thereby regulating their subcellular localization [15,21,22]. Additionally, the N-terminus contains a conserved cysteine-rich cluster, whose self-palmitoylation is sufficient to promote PM association [15,23]. Despite these insights, the mechanisms by which ABHD17 proteins are targeted to specific membranes and how their own palmitoylation state regulates their localization and activity remains incompletely understood.

Focusing on ABHD17A, we define a palmitoylation code within the N-terminal cysteine cluster that regulates intracellular distribution and PM targeting. Our findings demonstrate that (i) N-terminal palmitoylation is essential for PM association, (ii) palmitoylation of the middle cysteine pair serves as the principal determinant for PM targeting and catalytic activity, (iii) hydrophobic residues adjacent to the palmitoylation cluster facilitate an initial, pre-palmitoylation engagement with endomembranes, and (iv) tyrosine-based YXXØ (Y = tyrosine, X = any amino acid, Ø = bulky hydrophobic amino acid) motifs within the alpha/beta hydrolase fold mediate endosomal sorting consistent with recognition by AP-1 at the trans-Golgi network. Together, these findings delineate a stepwise mechanism that links site-specific N-terminal palmitoylation to ABHD17A trafficking and compartmentalized activity.

## 2. Results

### 2.1. N-Terminal Palmitoylation of the ABHD17 Family Is Essential for PM Localization

**Figure 1:**
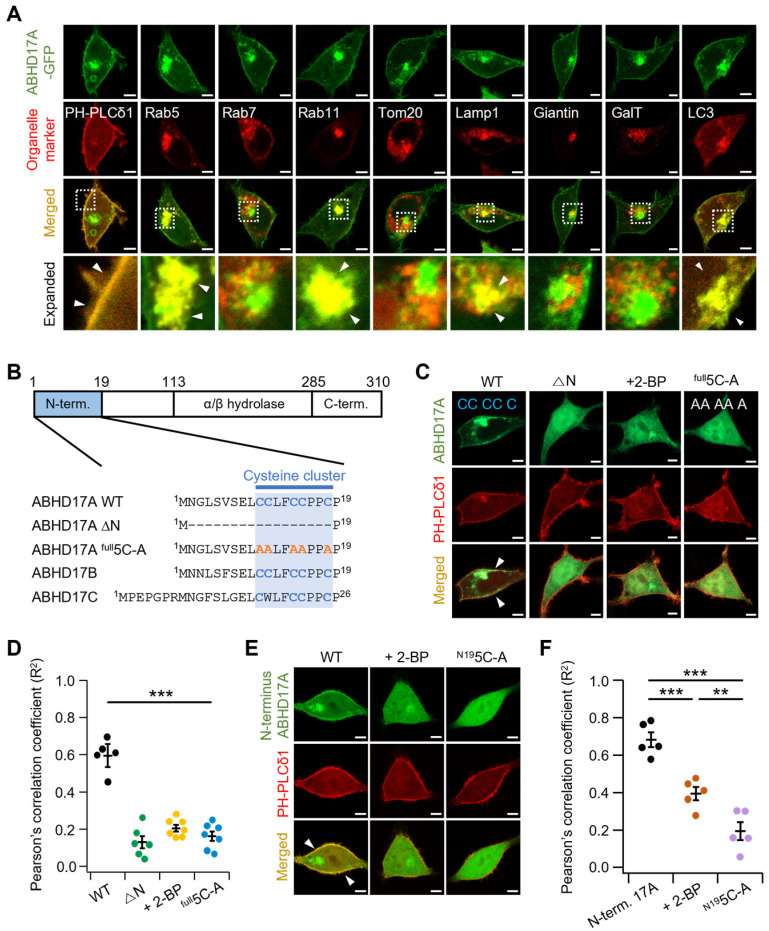
Subcellular localization of ABHD17A and requirement for N-terminal palmitoylation in HEK293T cells. (**A**) Representative confocal images of HEK293T cells expressing wild-type (WT) ABHD17A-GFP (green) alongside organelle markers (red): PH-PLCδ1 (PM), Rab5 (early endosome), Rab7 (late endosome), Rab11 (recycling endosome), Tom20 (mitochondria), Lamp1 (lysosome), Giantin (medial- and cis-Golgi), GalT (trans-Golgi network), and LC3 (autophagosome). The expanded images are indicated by dashed rectangles in the merged images. White arrowheads indicate co-localization between GFP and RFP. Scale bars: 5 μm. (**B**) Domain schematic of ABHD17A, showing the N-terminus, α/β-hydrolase domain, and C-terminus, along with alignment of the N-terminal cysteine clusters from ABHD17A/B/C. Cysteines in the cluster are highlighted in blue. Full-length ABHD17A-GFP (WT), deletion of residues 2–18 (△N), and all five N-terminal cysteines mutated to alanine (^full^5C-A; orange A indicates alanine substitutions). (**C**,**E**) Representative confocal images of cells expressing ABHD17A constructs (green) with PH-PLCδ1 (red). Isolated N-terminus (N1-19) and its palmitoylation deficit mutant (^N19^5C-A) derivative were used in (**E**). Some cells were incubated with 50 μM 2-BP for 12–24 Fh. White arrowheads indicate co-localization between GFP and RFP. Scale bars: 5 μm. (**D**,**F**) Quantification of colocalization between ABHD17A signals and PH-PLCδ1 was performed on whole-cell ROIs using Pearson’s correlation coefficient (R^2^) values (*n* = 5–7; each dot represents one cell). Data are mean ± SEM. **, *p* < 0.01, ***, *p* < 0.001, one-way ANOVA followed by Dunnett’s post hoc test.

ABHD17 family proteins are broadly expressed in vertebrates and localize to the PM and endosomal compartments. We examined their subcellular localization in more detail in HEK293T cells using various endomembrane markers. Consistent with prior reports, ABHD17 proteins localized to the PM, early endosomes (EEs), and recycling endosomes (REs) [15,22,23], and also to lysosomes and autophagosomes (Figure 1A and Figure S1A,B). To test whether palmitoylation determines this distribution, we first genetically engineered two ABHD17A variants that abolish N-terminal palmitoylation: a deletion lacking the N-terminal region (residues 2–18; ∆N) and a mutant with alanine at all five N-terminal cysteines (5C-A) (Figure 1B). Additionally, we treated cells expressing WT ABHD17A with the commonly used palmitoylation inhibitor 2-bromopalmitate (2-BP), verified using the PM marker pleckstrin homology domain of phospholipase C-δ1 (PH-PLCδ1). As shown in Figure 1C,D, the ABHD17A ∆N and 5C-A variants failed to associate with the PM, and treatment with 2-BP decreased ABHD17A palmitoylation and reduced PM association. Similar results were obtained for ABHD17B and ABHD17C (Figure S2). Consistent phenotypes were observed in HeLa cells, where disruption of N-terminal palmitoylation similarly abolished PM association, indicating that this requirement generalizes across cell types (Figure S3). These observations indicate that N-terminal palmitoylation is required for PM localization of ABHD17A, in line with previous work [15,23]. Interestingly, when expressed in isolation, the N-terminus of ABHD17A was sufficient to target the PM but did not localize to early or recycling endosomes (Figure 1E,F and Figure S4). Compared to full-length ABHD17A, the N-terminus displayed a partial cytosolic signal. Taken together, these data show that N-terminal palmitoylation is essential for localization to the PM, whereas sequences outside the N-terminus are required for trafficking to early and recycling endosomes.

### 2.2. Single N-Terminal Cysteine Codes Are Insufficient for ABHD17A Palmitoylation and Membrane Association

**Figure 2:**
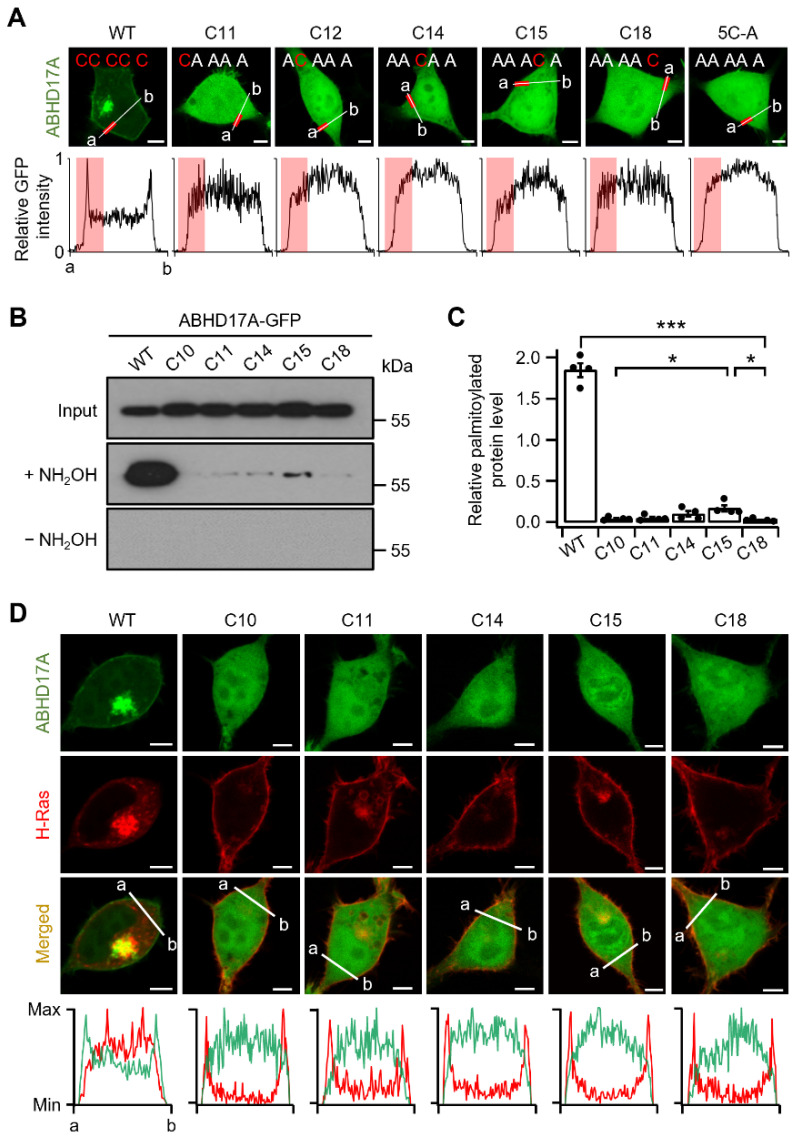
Single cysteines are insufficient for ABHD17A palmitoylation and PM targeting. (**A**) Representative confocal fluorescence images of cells expressing ABHD17A-GFP (WT) or single-cysteine variants that retain only one cysteine in the N-terminal cluster: C10-only (C11A/C14A/C15A/C18A), C11-only (C10A/C14A/C15A/C18A), C14-only (C10A/C11A/C15A/C18A), C15-only (C10A/C11A/C14A/C18A), C18-only (C10A/C11A/C14A/C15A), and ^full^5C-A. Line-scan plots below each image show normalized fluorescence intensity along the indicated a-b line. The red segment on the a-b line marks the PM region near point a, and the red shading in the line-scan highlights the same segment. Scale bars: 5 μm. (**B**) Acyl-RAC assay of palmitoylated ABHD17A from HEK293T cells expressing GFP-fused WT or single-cysteine variants. Equal volumes of protein samples treated with hydroxylamine (+) or control (−) were analyzed via SDS-PAGE and immunoblotting using anti-GFP antibodies. (**C**) Quantification of the Acyl-RAC assay normalized to input and expressed relative to WT (*n* = 4). Data are the mean ± SEM. *, *p* < 0.05, ***, *p* < 0.001, determined using one-way ANOVA followed by Dunnett’s post hoc test. (**D**) Representative confocal images of cells expressing ABHD17A-GFP (WT or single-cysteine variants) and mCherry-H-Ras. Line-scan plots along the a-b line show ABHD17A (green) and H-Ras (red) intensities. Scale bars: 5 μm.

We next examined whether individual cysteines within the N-terminal palmitoylation cluster differentially contribute to stable membrane association and palmitoylation. To this end, we generated single-cysteine variants that retain only one cysteine in the cluster (C10, C11, C14, C15, C18) and expressed them in HEK293T cells to assess intracellular distribution. Confocal imaging with normalized line scan analysis showed that all five single-cysteine variants, similar to the 5C-A mutant, failed to localize to the PM and to any endomembrane compartments (Figure 2A). Furthermore, palmitoylation was assessed by Acyl-RAC, an enrichment assay that selectively captures S-acylated cysteines after hydroxylamine treatment, thereby reporting palmitoylation levels [24]. Compared with ABHD17A WT, palmitoylation was not effectively detected in any of the single-cysteine variants (Figure 2B,C). These results indicate that a single N-terminal cysteine is insufficient for palmitoylation and for stable membrane association of ABHD17A, which harbors a multi-cysteine palmitoylation cluster. To test whether any single-cysteine variant retains depalmitoylase activity, we co-expressed mCherry-H-Ras, a well-established ABHD17A substrate. H-Ras contains two palmitoylation sites at its C-terminus (C181 and C184), and mCherry was fused at the N-terminus to avoid interference. In cells expressing ABHD17A WT, mCherry-H-Ras was depalmitoylated and released from the PM into the cytosol. In contrast, each single-cysteine variant failed to induce H-Ras depalmitoylation, indicating a loss of depalmitoylation activity, as shown in single-cell images and corroborated by multicell fields (Figure 2D and Figure S5). Collectively, these results demonstrate that ABHD17A requires more than one N-terminal cysteine (at least two) for S-palmitoylation, membrane association, and depalmitoylase activity.

### 2.3. Middle Region Palmitoylation in ABHD17A Is Necessary for PM Localization and Catalytic Activity

**Figure 3:**
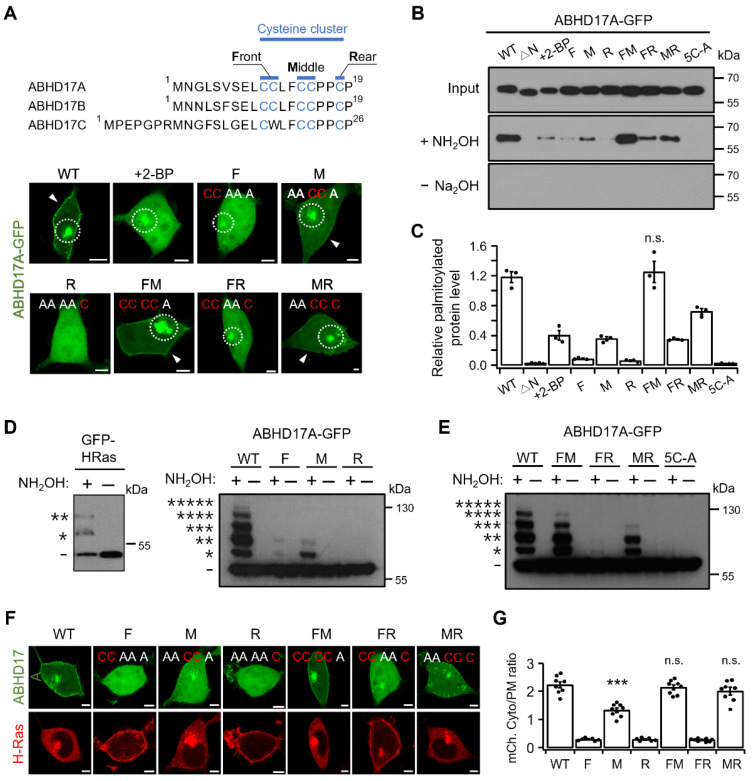
Palmitoylation in the middle region of ABHD17A is critical for their PM localization and depalmitoylation activity. (**A**) Top, organization of the N-terminal cysteine cluster in ABHD17 family members. Front (F) = C10/C11, Middle (M) = C14/C15, Rear (R) = C18. Bottom, representative confocal images of HEK293T cells expressing ABHD17A-GFP (WT) or the indicated code constructs (F, M, R, FM, FR, MR). Where indicated, cells were treated with 50 μM 2-BP for 12–24 h. Dotted circles mark intracellular accumulations, and arrowheads indicate PM signals. Inset sequence labels denote retained cysteines as “C” and alanine substitutions as “A”. Scale bars: 5 μm. (**B**) Acyl-RAC assay of palmitoylated ABHD17A from cells expressing GFP-fused WT or code constructs, with WT + 2-BP (50 μM, 12–24h) as a pharmacological control. Equal volumes of protein samples treated with hydroxylamine (+) or control (−) were analyzed by SDS-PAGE and immunoblotting using anti-GFP antibodies. (**C**) Quantification of Acyl-RAC assay normalized to input and expressed relative to WT (*n* = 3) Data are mean ± SEM. Differences were considered not significant (n.s.). (**D**) Acyl-PEG exchange (APE) was performed to resolve the number of modified cysteines. Left, GFP-H-Ras positive control, Right, ABHD17A WT and single-region constructs (F, M, R). Equal volumes of protein samples treated with hydroxylamine (+) or negative control (−) were analyzed by SDS-PAGE and immunoblotting using anti-GFP antibodies. The number of asterisks (*) indicate successive PEG-induced mass shifts corresponding to the number of palmitoylated cysteines; absence of an asterisk denotes zero shift. (**E**) APE results for WT and paired-region constructs (FM, FR, MR) along with the 5C-A negative control. The number of asterisks (*) indicate successive PEG-induced mass shifts corresponding to the number of palmitoylated cysteines; absence of an asterisk denotes zero shift. (**F**) Representative confocal images of cells expressing ABHD17A-GFP (WT or constructs) and mCherry-H-Ras. Scale bars: 5 μm. (**G**) Quantification of the mCherry-H-Ras fluorescence ratio (Cyto/PM) from (**F**) (*n* = 6–8; each dot represents one cell). Data are mean ± SEM. ***, *p* < 0.001; n.s., not significant, with one-way ANOVA followed by Dunnett’s post hoc test.

ABHD17A contains multiple N-terminal cysteines that can be differentially palmitoylated. Consequently, the protein can exist in non-, mono-, and multi-palmitoylated states. Previous studies have suggested that multisite palmitoylation can encode intracellular patterning and function [17,18,19]. We therefore investigated whether defined subsets of N-terminal cysteines (hereafter, codes) program ABHD17A palmitoylation states, intracellular distribution, and PM targeting. To restrict palmitoylation to defined positions within the N-terminal cluster, we generated alanine-substitution mutants and denote them as follows: F retains C10 and C11 (C14A/C15A/C18A); M retains C14 and C15 (C10A/C11A/C18A); R retains C18 (C10A/C11A/C14A/C15A); FM (C18A); FR (C14A/C15A); MR (C10A/C11A). When expressed in HEK293T cells, ABHD17A WT, M, FM, and MR localized to the PM and to early and recycling endosomes, whereas F, R, and FR which lack an intact M region, did not localize to the PM (Figure 3A). The F construct exhibited endosomal localization without PM association, and R alone showed no detectable targeting to any compartments. However, adding F or M (FR or MR) restored endosomal localization and PM association. This difference between F and R likely reflects the unequal number of retained palmitoylation sites. Notably, the M construct partially colocalized with the trans-Golgi network and mitochondria (Figure S6). By contrast, WT, FM, and MR showed no detectable colocalization in these compartments, consistent with altered sorting at the trans-Golgi network when F or R are present, and with reduced residence or diversion to other compartments. As with WT, FM exhibited lysosomal localization (Figure S7). Taken together, the imaging data identify the M region as the principal determinant of PM targeting, with the F and R subsets modulating endosomal routing.

Palmitoylation was then quantified by Acyl-RAC assay. The FM construct exhibited palmitoylation levels comparable to WT, whereas F and R were shown to be weakly palmitoylated. Inclusion of the M region significantly increased palmitoylation levels in FM and MR. Treatment with 2-BP and the 5C-A mutant were used as negative controls. To determine the palmitoylation stoichiometry of ABHD17A, we performed Acyl-PEG exchange (APE) experiments. The APE assay reports the number of palmitoylations by tagging a 5 kDa mass tag onto hydroxylamine-exposed cysteines, producing discrete upward shifts in apparent molecular mass [25]. It is important to note that accurate quantification can be challenging for variable and low-quantity proteins, as the APE assay is less sensitive than the Acyl-RAC assay. As a control, GFP–H-Ras produced two discrete mass-shifted bands (the number indicated by * represents the number of residues that are palmitoylated in each band), consistent with its two C-terminal palmitoylation sites (Figure 3D). WT ABHD17A exhibited a stepwise ladder of bands corresponding to zero through five palmitoylations, with a band at the highest mass shift (five sites) which was detectable but faint. Band intensities were not uniform, with the two-site palmitoylated form was relatively strong, while the four-site palmitoylated form was comparatively weak. The FM construct showed shifts up to the fourth band, the MR construct up to the third band, and the R construct showed no shifted bands. Inclusion of the M region increased the maximum shift and overall ladder complexity (FM, MR) (Figure 3D,E). These APE results, together with the confocal imaging and Acy-RAC data, indicate that the M region is the principal driver of multisite palmitoylation in ABHD17A and a key determinant of its endosomal routing and PM localization.

We next assessed depalmitoylase function using H-Ras. In HEK293T cells co-expressing mCherry-H-Ras, constructs containing the M region (WT, M, FM, MR) promoted depalmitoylation and facilitated the release of H-Ras from the PM into the cytosol, whereas constructs F and R did not show this effect (Figure 3F,G and Figure S8). Similarly, only M-containing ABHD17C constructs were able to release H-Ras from the PM (Figure S9). It is important to note that this does not imply that the M region itself acts directly on H-Ras. Rather, the data suggest that ABHD17A must undergo self-palmitoylation via the M region and reach the PM in order to effectively act on its membrane-bound substrate. Altogether, our results indicate that the M region is critical for both the multisite palmitoylation of ABHD17A and its catalytic activity at the PM. Furthermore, palmitoylation codes regulate trafficking, thereby shaping intracellular localization and consequent function.

### 2.4. Hydrophobic Residues in the N-Terminus Regulate Initial Endomembrane Engagement Prior to Palmitoylation

**Figure 4:**
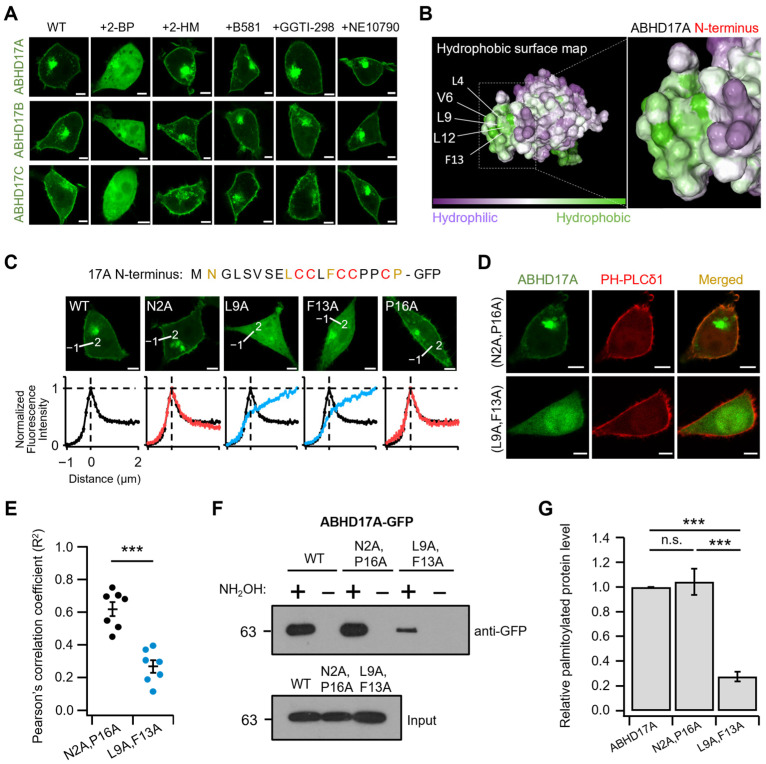
Hydrophobic N-terminal residues enable initial endomembrane engagement. (**A**) Representative confocal images of HEK293T cells expressing ABHD17 family members treated with 2-BP (50 μM), 2-HM (100 μM), B581 (100 μM), GGTI-298 (10 μM), or NE 10790 (200 μM) for 12–24h. Scale bars: 5 μm. (**B**) Solubility surface map of ABHD17A viewed from the N-terminal side (left) and a magnified view of the N-terminal segment (right). Hydrophobic patches are shown in green, and selected residues (L4, V6, L9, L12, F13) are indicated. (**C**) Top, ABHD17A N-terminal sequence (N-19) with palmitoylated cysteines highlighted in red and residues mutated to alanine in the indicated constructs (N2A, L9A, F13A, P16A) shown in dark yellow. Middle, representative confocal images for the N-terminal sequence of ABHD17A and its mutants. Scale bars: 5 μm. Bottom, normalized line-scan profiles taken along the white line in the images. Red traces correspond to N2A and P16A, blue traces to L9A and F13A, and black traces to WT. On the x-axis, 0 indicates the PM. (**D**) Representative confocal images of cells expressing ABHD17A mutants (N2A/P16A, L9A/F13A) with RFP-PH-PLCδ1. Scale bars: 5 μm. (**E**) Pearson’s correlation coefficient (R2) values between ABHD17A constructs and PH-PLCδ1 (*n* = 7). Data are mean ± SEM. ***, *p* < 0.001, one-way ANOVA followed by Dunnett’s post hoc test. (**F**) Acyl-RAC assay of ABHD17A-GFP, N2A/P16A, and L9A/F13A mutants. Equal volumes of protein samples treated with hydroxylamine (+) or control (−) were analyzed by SDS-PAGE and immunoblotting using anti-GFP antibodies. (**G**) Quantification of Acyl-RAC normalized to input and expressed relative to WT (*n* = 5). Data are the mean ± SEM. ***, *p* < 0.001, n.s., not significant, with one-way ANOVA followed by Dunnett’s post hoc test.

To define the step preceding palmitoylation, we examined how ABHD17A first engages with endomembranes where zDHHC-PATs reside. Prior work [15,23], along with our observations, indicates that this short N-terminal segment is sufficient to support PM targeting and to prime ABHD17A for subsequent palmitoylation. We therefore evaluated the contribution of other lipid modifications to this initial step using a panel of inhibitors: 2-BP, 2-hydroxymyristic acid (2-HM; N-myristoylation inhibitor), B581 (farnesylation inhibitor), GGTI-298 (geranylgeranylation I inhibitor), and NE10790 (geranylgeranylation II inhibitor). Only 2-BP significantly altered ABHD17A localization, markedly reducing PM association, while inhibition of N-myristoylation and prenylation (farnesylation and geranylgeranylation) yielded localization patterns comparable to WT (Figure 4A). The N-terminus of ABHD17A also exhibited inhibitor sensitivity similar to that of WT (Figure S10A). These results indicate that robust PM association of ABHD17A depends on palmitoylation, and we observed no contribution of N-myristoylation or prenylation to the early endomembrane engagement step.

We next used AlphaFold2 to model the fold of full-length ABHD17A and Protein-Sol to predict its solubility. Guided by the solved structure of APT1, a representative alpha/beta hydrolase, the ABHD17A model was interpreted using APT1 as a structural benchmark to contextualize the overall fold [26]. The N-terminus of ABHD17A exhibited pronounced hydrophobic character (Figure 4B), whereas the predicted electrostatics were approximately neutral according to Protein-Sol (Figure S10B). These biophysical features prompted us to test whether hydrophobic residues adjacent to the N-terminal palmitoylation cluster facilitate initial endomembrane engagement and PM association. Thus, we performed alanine-scanning mutagenesis of the N-terminus-GFP construct. Confocal imaging with line scanning analysis revealed that single substitutions at L4, V6, L9 and F13 reduced PM association. The effects were modest for L4A and V6A, which retained partial PM signal, while L9A and F13A nearly abolished PM localization (Figure 4C and Figure S11). Despite the powerful application of alanine scanning, we created double mutant forms (L9A/F13A and N2A/P16A as a negative control) to account for potential cooperative interactions between residues. Interestingly, the L9A/F13A double mutant not only lacked colocalization with the PM marker PH-PLCδ1, but it also exhibited almost no ability to reach the endomembrane. In contrast, the randomly mutated negative control double mutant N2A/P16A was well expressed in both the PM and endomembrane (Figure 4D,E). Finally, Acyl-RAC confirmed a marked reduction in palmitoylation for the L9A/F13A double mutant relative to WT ABHD17A (Figure 4F,G). Taken together, these data indicate that hydrophobic residues in the N-terminus, particularly L9 and F13, promote the pre-palmitoylation endomembrane engagement that enables subsequent palmitoylation and proper PM targeting.

### 2.5. Endosomal Targeting Motif in ABHD17A Contributes to PM Targeting

**Figure 5:**
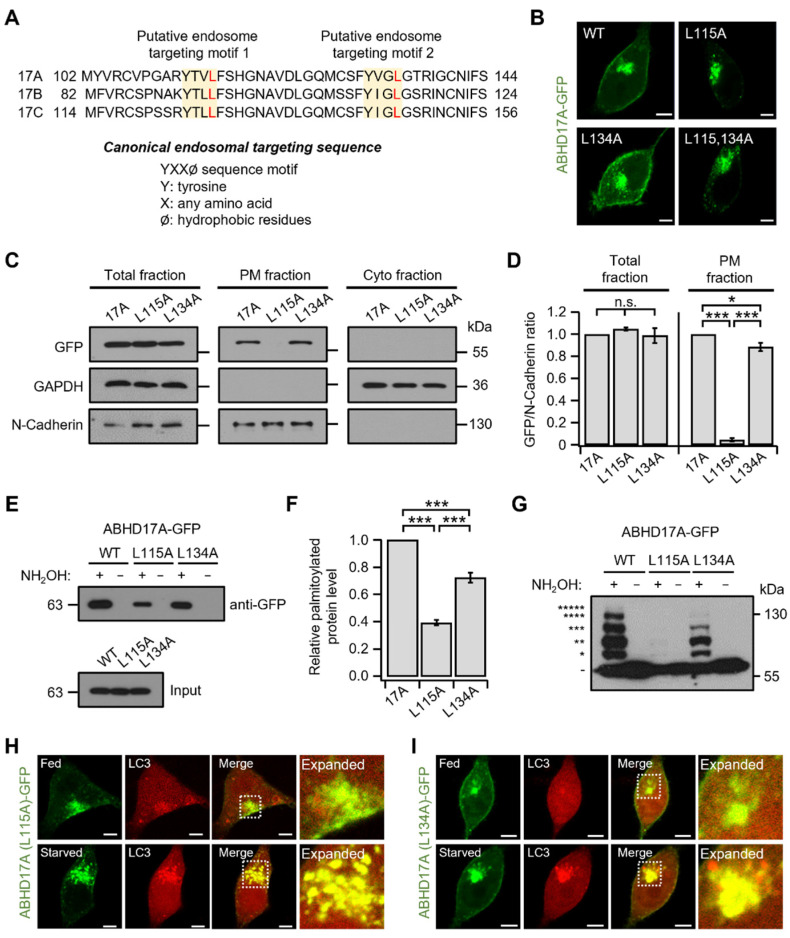
Tyrosine-based endosomal sorting motifs in ABHD17A regulate palmitoylation and PM delivery. (**A**) Sequence segments of the ABHD17 family showing two putative YXXØ endosomal sorting motifs within the alpha/beta-hydrolase fold. Tyrosine (Y) and the hydrophobic Ø position (Leu) are highlighted. A consensus YXXØ sequence is shown below. (**B**) Representative confocal images of cells expressing ABHD17A-GFP, L115A, L134A, or the double mutant L115/134A. Scale bars: 5 μm. (**C**) Immunoblot analysis of total lysates, PM fractions, and cytosolic fractions from cells expressing ABHD17A, L115A, or L134A. Expression levels were detected using anti-GFP, N-cadherin and GAPDH serving as markers for PM and cytosol fractions, respectively. (**D**) Quantification of the GFP signal normalized to N-Cadherin in the PM fraction, with total lysate shown for reference (*n* = 3). Data are the mean ± SEM. *, *p* < 0.05, ***, *p* < 0.001, n.s., not significant, with one-way ANOVA followed by Dunnett’s post hoc test. (**E**) Acyl-RAC assay of ABHD17A-GFP, L115A, and L134A. Equal volumes of protein samples treated with hydroxylamine (+) or control (−) were analyzed by SDS-PAGE and immunoblotting using anti-GFP antibodies. (**F**) Quantification of the Acyl-RAC normalized to input (*n* = 4). Data are mean ± SEM. ***, *p* < 0.001, with Student’s two-tailed unpaired *t*-test. (**G**) APE assay for ABHD17A-GFP, L115A, and L134A. The number of asterisks (*) indicate successive PEG-induced mass shifts corresponding to the number of palmitoylated cysteines; absence of an asterisk denotes zero shift. Equal volumes of protein samples treated with hydroxylamine (+) or negative control (−) were analyzed by SDS-PAGE and immunoblotting using anti-GFP antibodies. Asterisks indicate successive PEG-induced mass shifts; absence of asterisk denotes zero shift. (**H**,**I**) Colocalization of ABHD17A mutants with the autophagosome marker LC3 under nutrient-replete (Fed) and serum-starved (for 2–4 h before imaging) conditions. Scale bars: 5 μm.

In eukaryotic cells, membrane proteins are synthesized on ER-bound ribosomes and traffic through the Golgi to the PM or intracellular organelles [27,28]. As proteins are routed to their functional destinations, precise subcellular localization underpins protein function and compartment-specific interactions [29]. We therefore sought to identify the sequence determinants that direct ABHD17A to specific compartments. As shown in Figure 1, the N-terminus of ABHD17A was sufficient for PM targeting and lysosomal localization but did not localize to early or recycling endosomes, unlike full-length ABHD17A. This divergence suggested that sequences outside the N-terminus mediate endosomal targeting. Accordingly, sequence analysis of ABHD17A revealed two putative tyrosine-based YXXØ motifs within the alpha/beta hydrolase fold as candidate endosomal sorting signals (Figure 5A), with the local structure of Tyrosine perhaps stabilized by Ar-based intramolecular interactions, as has been established [30]. The tyrosine-based YXXØ motif serves as a canonical endosomal targeting sequence, recognized by the adaptor protein complex 1 (AP-1), promoting shuttling between the trans-Golgi network and endosomes [31,32]. To determine whether the two YXXØ motifs influence the intracellular expression of ABHD17A, leucine residue at Ø was substituted with alanine (L115A and L134A), respectively, and confocal microscopy was performed. L115A caused a marked loss of ABHD17A at the PM and reduced the signal at endosomes and the Golgi, whereas L134A maintained clear PM localization while also forming intracellular puncta (Figure 5B and Figure S12).

Additionally, we quantified PM abundance by biochemical PM–cytosol fractionation followed by immunoblotting. WT and L134A were readily detected in the PM fraction, but L115A was not, despite similar total expression and cytosolic levels across constructs (Figure 5C,D). These findings suggest that L115A is properly synthesized yet fails to reach or be retained at the PM. To corroborate these findings, we performed Acyl-RAC and APE assays. The palmitoylation level of L115A was significantly decreased (~60%), and we observed only faint shifts of one or two, with higher-order bands absent. In contrast, L134A exhibited a modest ~20% decrease in palmitoylation, yet bands up to the fourth band were detected, with the four-site band faint but detectable (Figure 5E–G). Consistent with a diversion to degradative pathways, imaging with LC3 revealed preferential accumulation of endosomal-motif mutants in autophagosomes. Under nutrient-replete (Fed) conditions, LC3 puncta were scarce, and colocalization with ABHD17A constructs was minimal. Upon serum starvation (starved), L115A exhibited near complete colocalization with LC3 puncta, while L134A also partial colocalized (Figure 5H,I). In contrast to the broader distribution of WT ABHD17A across the PM, early and recycling endosomes, and lysosomes, with some overlap with LC3 puncta (Figure 1A), LC3 imaging indicated that the endosomal motif mutants preferentially accumulated on LC3-positive autophagosomes. The motif 1 mutant L115A showed the strongest shift, and no detectable enrichment was observed at other endosomal or Golgi markers (Figure S12). Analogous tyrosine-based motif mutants of ABHD17B (L95A, motif 1; L114A, motif 2) and ABHD17C (L127A, motif 1; L146A, motif 2) recapitulated these phenotypes, including reduced PM targeting, and enhanced LC3 colocalization upon serum starvation (Figure S13). Collectively, these findings support a model in which tyrosine-based YXXØ motifs, particularly motif 1 (L115), mediate an endosomal sorting step consistent with AP-1 recognition at the trans-Golgi network that is required for efficient multisite palmitoylation and subsequent delivery to the PM, and disruption of this process reduces palmitoylation and diverts ABHD17A to autophagosomes.

## 3. Discussion

**Figure 6:**
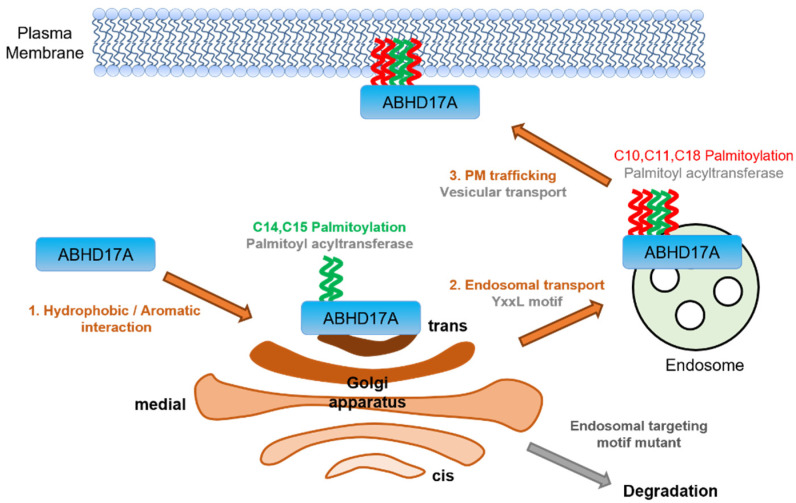
Stepwise model for PM targeting of ABHD17A. Schematic of the proposed itinerary derived from this study. (1) Pre-palmitoylation capture: hydrophobic/aromatic residues adjacent to the N-terminal cysteine cluster mediate the initial engagement of ABHDA17A with endomembranes that contain zDHHC-PATs. (2) Middle-region palmitoylation and sorting: self-palmitoylation on the middle cysteine pair C14/C15 (green) occurs at the trans-Golgi network, followed by YXXØ-dependent sorting to endosomes. (3) Completion and delivery: additional palmitoylation at the front and rear sites C10/C11 and C18 (red) enables vesicular transport to the PM, where ABHD17A acts on membrane-bound substrates. Disruption of the YXXØ motif or weakening of the initial hydrophobic engagement reduces multisite palmitoylation, impairs PM delivery, and diverts ABHD17A toward autophagosomal degradation. Illustration created in Microsoft PowerPoint (Microsoft Office Professional Plus 2019, Version 1808).

This study defines a multi-step itinerary by which the depalmitoylase ABHD17A achieves PM residency and catalytic activity. We demonstrate that site-specific palmitoylation within a conserved N-terminal cysteine cluster functions as a palmitoylation “code” that dictates intracellular distribution and function. In this code, modification of the middle region (C14, C15) serves as the principal determinant for PM targeting and activity toward membrane-bound substrates such as H-Ras, whereas the front (C10, C11) and rear (C18) positions modulate endosomal routing, thereby influencing delivery to the PM (Figure 6). These findings support the broader concept that multisite palmitoylation can encode distinct trafficking outcomes for a single protein, as established for SNAP25 and the PI4KIIIα complex [17,18]. Additionally, they align with emerging perspectives that Golgi electrostatics, including PtdIns4P-dependent negative charge, play a crucial role in recruiting and positioning substrates, and that thioesterases actively shape palmitoylation cycles by engaging membranes and extracting acyl chains [19,20].

Our mutational analysis demonstrates functional non-equivalence among the five N-terminal cysteines. Constructs that retain the middle region supported PM association, exhibited higher overall palmitoylation, and enhanced depalmitoylation of H-Ras, whereas constructs lacking the middle region failed in all three respects. The APE ladder for wild-type ABHD17A revealed species with zero to five modified cysteines, with a faint band corresponding to the five-site band, indicating a continuum of palmitoylation states whose distribution is influenced by the palmitoylation code. This organization resembles consecutive cysteine arrays in other multiply palmitoylated proteins, where local clustering promotes stable membrane engagement and can tune function [33]. Moreover, live-cell imaging revealed that depalmitoylated Ras dynamically shuttles the PM and the Golgi through a nonvesicular route, indicating continuous turnover of its palmitoylation state [34]. Complementary studies showed that the palmitoylation and depalmitoylation cycle actively regulates the localization and signaling activity of palmitoylated Ras isoforms, underscoring the dynamic nature of this modification [35]. These dynamics are consistent with our APE results, which indicate that ABHD17A exists in multiple palmitoylation states and likely traffics within cells through an ongoing cycle of palmitoylation and depalmitoylation.

Which zDHHC isoforms install these modifications remains to be determined. Several zDHHC enzymes localize to distinct organelles, including reported pools at the PM and the Golgi, as well as others in the endoplasmic reticulum. This localization suggests that ABHD17A may encounter different acyltransferases along its trafficking route. Furthermore, multiple studies in mammalian systems indicate that individual substrates can be palmitoylated by more than one zDHHC enzyme, highlighting overlapping substrate specificities within the family [36,37,38]. It also remains unclear whether the front and rear cysteines are modified in endosomes, at the PM, or in both locations.

An early prerequisite in this pathway is endomembrane engagement at membranes where zDHHC-PATs reside. Since ABHD17A lacks transmembrane segments, it must first engage these membranes to access the palmitoylation machinery. Alanine scanning revealed that hydrophobic residues adjacent to the cysteine cluster, most notably L9 and F13, are critical for this initial engagement, as double substitution diminished both membrane association and S-acylation. Inhibitor profiling indicated that other lipidation pathways do not contribute at this step; blocking N-myristoylation or prenylation did not alter the localization of ABHD17A, in contrast to Ras, which requires farnesylation before palmitoylation can occur [39]. Together with the Golgi electrostatics model, these observations support a view in which hydrophobic side chains cooperate with local charged surfaces to capture ABHD17A prior to acylation, facilitating productive encounters with zDHHCs [19].

We also identify tyrosine-based YXXØ motifs within the alpha/beta hydrolase fold as endosomal sorting signals required to complete the itinerary. Disruption of motif 1 (L115A) reduced surface abundance, lowered overall S-acylation, and prevented efficient delivery to the PM, while disruption of motif 2 (L134A) resulted in a milder phenotype, perhaps due in some part to the loss of stabilizing Ar-based intramolecular interactions stabilizing the local structure; the latter could be explored through homology sequencing with Phe and Trp replacing the Tyrosyl residues to probe this [30]. Although YXXØ signals can mediate internalization that is dependent on AP-2 [40], the loss of motif 1 decreased, rather than increased, PM levels. This pattern argues against an AP-2–driven endocytic route and is consistent with recognition by AP-1 at the interface between the trans-Golgi network and endosomes. The requirement for endosomal passage explains why the isolated N-terminus, although sufficient for partial PM targeting, does not reflect the full trafficking spectrum of the intact protein. It also provides a mechanistic link between the palmitoylation code and compartmental sorting.

Autophagosome imaging further contextualizes the trafficking defects. Under nutrient-replete conditions, LC3 puncta were scarce, and colocalization with ABHD17A constructs was minimal. Upon serum starvation, the L115A mutant exhibited near-complete overlap with LC3, and the L134A mutant also showed colocalization to a lesser extent. These data suggest that the failure to execute endosomal sorting and multisite palmitoylation can divert ABHD17A into autophagic sequestration or quality control pathways, rather than indicating that ABHD17A induces autophagy. The partial LC3 overlap observed for wild-type ABHD17A may reflect basal turnover of intermediate acylation states, a possibility that warrants direct testing, especially considering that PPT1 is a lysosomal thioesterase that depalmitoylates S-acylated proteins in lysosomes prior to their degradation [41,42].

Endogenous ABHD17 family display isoform redundancy in HEK293T cells, and pronounced effects on Ras palmitate turnover often require combined isoform depletion [15]. Consistent evidence from other human systems shows that selective ABHD17 inhibition (ABD957) and CRISPR deletion of ABHD17A/B modulate N-Ras palmitoylation and signaling [21], and that attenuation of ABHD17 increases palmitoylation PM localization of NOD2 [43]. Because our mutant phenotypes interrogate cis-encoded localization and sorting rules of ABHD17A, reducing endogenous ABHD17 is not expected to re-route mutant localization, although multiplex knockdown would be predicted to further affect substrate depalmitoylation kinetics. Future studies using multiples KD/KO with rescue will be valuable to extend these findings under reduced endogenous ABHD17.

In summary, we propose a stepwise mechanism in which hydrophobic residues adjacent to the N-terminal cluster initiate endomembrane engagement. The middle cysteine region undergoes palmitoylation at the trans-Golgi network, while YXXØ motifs mediate an endosomal sorting step. Additional palmitoylation at the front and rear sites completes the delivery to the PM. This itinerary links site-specific palmitoylation to compartmentalized activity, explaining how ABHD17A reaches the PM to depalmitoylate substrates such as H-Ras. Consistent with this framework, analogous experiments with ABHD17B and ABHD17C demonstrated the same dependence on the middle region, suggesting that palmitoylation codes are conserved across the ABHD17 family and may extend to other depalmitoylases. By establishing how a depalmitoylase is positioned by a palmitoylation code, these findings extend the paradigm of multisite S-acylation from cargo proteins to enzymes that regulate the palmitoylation cycle.

## 4. Materials and Methods

### 4.1. Cell Culture and Transfection

HEK293T cells were obtained from Bertil Hille (University of Washington School of Medicine, Seattle, WA, USA), and HeLa cells were obtained from the Korean Cell Line Bank (Seoul National University, Seoul, Republic of Korea). Both cell lines were cultured in DMEM (HyClone, Logan, UT, USA) supplemented with 10% FBS (HyClone, Logan, UT, USA) and 0.2% penicillin/streptomycin (HyClone, Logan, UT, USA) at 37 °C and 5% CO_2_. Cells were transfected with various cDNAs using Lipofectamine 2000 (Invitrogen, Waltham, MA, USA) according to the manufacturer’s instructions. For confocal microscopy experiments, cells were plated onto coverslip chips coated with 0.1 mg/mL poly-L-lysine (Sigma-Aldrich, St. Louis, MO, USA) 24 to 36 h after transfection, and the cells were used within 12 to 24 h after seeding.

### 4.2. Western Blotting

For protein detection, samples were resolved on 8–10% SDS–PAGE and transferred to PVDF membranes (Bio-Rad, Hercules, CA, USA) using either wet or semi-dry transfer. Membranes were blocked in 5% (*w*/*v*) skim milk (Sigma-Aldrich, St. Louis, MO, USA) in TBS-T and incubated overnight at 4 °C with primary antibodies (Anti-GFP 1:1000, Cell signaling (Danvers, MA, USA), #2955; anti-N-cadherin 1:1000, BD bioscience (Franklin Lakes, NJ, USA), #610920; anti-GAPDH 1:10,000, Cell signaling, #2118). After thorough washes in TBS-T, membranes were incubated with HRP-conjugated secondary antibodies: anti-rabbit IgG (1:5000, Thermo (Waltham, MA, USA), #31460) and anti-mouse IgG (1:5000, Jackson Immuno Research (West Grove, PA, USA), #115-035-003). The membranes were then washed again and developed with ECL solution (SuperSignal™ West Pico PLUS Chemiluminescent substrate, Thermo, #34577; SuperSignal™ West Femto Maximum Sensitivity Substrate, Thermo, #34095). Chemiluminescent signals were captured on X-ray film, and band intensities were quantified using ImageJ (NIH) software (version 1.53q).

### 4.3. PM–Cytosol Separation

PM and cytosolic fractions were isolated using the Plasma Membrane Protein Extraction Kit (#ab65400, Abcam, Cambridge, UK) following the manufacturer’s protocol. Briefly, transfected HEK293T cells were washed with ice-cold DPBS, harvested, resuspended in kit’s homogenize buffer containing protease inhibitors. The cells were then disrupted on ice with a Dounce homogenizer. Lysates were cleared at low speed and processed through the kit’s phase-separation steps to obtain a PM–enriched fraction and a matched cytosolic fraction. Protein concentrations were determined using the BCA assay, and equal amounts of protein were analyzed by SDS–PAGE and immunoblotting.

### 4.4. In Silico Analyses

An AlphaFold protein structure database (AlphaFoldDB) [44] model for rat ABHD17A (UniProt Q5XIJ5; AF-Q5XIJ5-F1-v4) served as a fold-level reference. The deposited model PDB was analyzed with Protein-Sol v2.0 (https://protein-sol.manchester.ac.uk/, accessed on 15 October 2024) to visualize sequence/structure-derived solubility and electrostatic properties of the N-terminus of ABHD17A. These predictions guided the selection of residues for alanine scanning. Guided by the solved structure of APT, a representative α/β-hydrolase, the ABHD17A model was interpreted using APT1 as a structural benchmark to contextualize the overall fold. The protein-Sol server estimates solubility and electrostatic property from sequence-based features calibrated to experimental datasets and reports per-residue profiles along the protein, as described previously [45].

### 4.5. Plasmids and Molecular Cloning

Rat ABHD17A cDNA (NM_001006983) was generously provided by Elizabeth Conibear (University of British Columbia, Vancouver, BC, Canada). The coding sequences for ABHD17B (NM_001438038.1) and H-Ras (NM_008284) were PCR-amplified from rat and mouse brain cDNA, respectively. Mouse ABHD17C cDNA was kindly provided by Masaki Fukatka (National Institute for Physiological Sciences, National Institutes of Natural Sciences, Okazaki, Japan). The PH-PLCδ1 biosensor (PH-RFP) was from Ken Mackie (University of Washington, Seattle, WA, USA). Constructs for Giantin, Rab5, Rab7, Rab11, and LAMP1 were sourced from Deok-Jin Jang (Kyungpook National University, Sangju, Republic of Korea).

ABHD17 family constructs were subcloned into the pEGFP-N1 vector (Clontech, Mountain View, CA, USA). H-Ras was PCR-amplified and inserted into the mCherry-C1 vector (Clontech, Mountain View, CA, USA). The N-terminal deletion (ΔN, residues 1–19) and all point-mutation variants were generated using inverse PCR. Single-cysteine variants were constructed as follows: C10-only (C11A/C14A/C15A/C18A), C11-only (C10A/C14A/C15A/C18A), C14-only (C10A/C11A/C15A/C18A), C15-only (C10A/C11A/C14A/C18A), and C18-only (C10A/C11A/C14A/C15A). “Code” mutants that restrict palmitoylatable positions within the N-terminal cluster included: F retains C10/C11 (C14A/C15A/C18A), M retains C14/C15 (C10A/C11A/C18A), R retains C18 (C10A/C11A/C14A/C15A), FM = C18A, FR = C14A/C15A, and MR = C10A/C11A. All constructs were verified by DNA sequencing (Macrogen, Republic of Korea).

### 4.6. Acyl-RAC Capture and Acyl-PEG Exchange Assays

Acyl-RAC and Acyl-PEG exchange experiments were performed using the commercially available CAPTUREomeTM S-Palmitoylated Protein Kit (Badrilla, Leeds, UK) and SiteCounterTM S-Palmitoylated Protein Kit (Badrilla, Leeds, UK), respectively, following the manufacturer’s protocols [24,25]. In brief, transfected HEK293T HEK293T cells were washed with DPBS, lysed in RIPA buffer, and total protein was adjusted to 1mg/mL as determined by the Pierce^TM^ BCA Protein Assay Kit (Thermo Fisher Scientific, #23227). Free thiols were blocked in Buffer A containing the Thiol Blocking Reagent at 40 °C for 4 h, followed by precipitation with three volumes of ice-cold acetone (−20 °C, 20 min) and five washes with 70% acetone. Pellets were re-dissolved in 1× binding buffer at 40 °C with shaking. An aliquot of soluble lysate (30 µL) was mixed with 2× Laemmli sample buffer and retained as the input fraction (IF). Samples were divided into thioester-cleaved (experimental) and acyl-preserved (negative control) conditions.

For Acyl-RAC, 50 µL of pre-equilibrated capture resin slurry was added to each tube. Freshly prepared thioester cleavage reagent or acyl-preservation reagent was added at 19 µL to experimental or control samples, respectively, and tubes were rotated for 2.5 h at room temperature. Resin was collected at 16,000× *g* for 1 min, washed five times with 1× binding buffer, eluted with 50 µL 2× Laemmli buffer, and heated at 60 °C for 10 min. For Acyl-PEG exchange, thioester cleavage reagent of acyl-preservation reagent was added at 11 µL to experimental or control samples, respectively, and incubated for 1 h at room temperature. The desalting spin columns were washed twice with 1× binding buffer (6500× *g*, 30 s each). Samples were applied and centrifuged at 6500× *g* for 1 min. The resulting eluate was incubated with 12 µL mass tag reagent for 1 h at room temperature. Samples from Acyl-RAC and APE were analyzed by SDS-PAGE as described.

### 4.7. Confocal Imaging and Quantitation

All imaging was performed using a Zeiss LSM 700 or LSM 800 confocal microscope (Carl Zeiss, Oberkochen, Germany) at room temperature (22–25 °C). The external solution contained 160 mM NaCl, 2.5 mM KCl, 1 mM MgCl_2_, 2 mM CaCl_2_, 10 mM HEPES, and 8 mM glucose, adjusted to pH 7.4 with NaOH, resulting in an osmolarity of 321–350 mOsm. Quantitative colocalization was performed in Fiji (ImageJ) using the Colocalization Threshold plugin to calculate Pearson’s correlation coefficients (R^2^). Analyses were computed on manually defined whole-cell ROIs with identical procedures across conditions, and each dot represents an independent cell. For the quantification of H-Ras redistribution, the ratios for cytosolic to PM fluorescence (Cyto/PM) and its inverse (PM/Cyto) were computed in ZEN. For each cell, five non-overlapping cytosolic regions of interest were randomly placed, and their mean fluorescent intensities averaged. PM intensity was obtained by drawing a line region of interest along the edge of the PM and calculating the mean intensity. Ratios were calculated per cell from these mean values. Image files were exported from the Zeiss LSM4 to JPEG format, and raw data were processed in Excel (Microsoft, Redmond, WA, USA) and Igor Pro (WaveMetrics, Portland, OR, USA). All schematic illustrations, including Figure 6, were created by the authors using Microsoft PowerPoint (Microsoft Office Professional Plus 2019, Version 1808).

### 4.8. Pharmacological Treatments

For pharmacological inhibition, transfected HEK293T cells were incubated with inhibitors for 12–24 h at 37 °C in 5% CO_2_ prior to imaging. The following agents were used: 2-bromopalmitate (2-BP, Sigma-Aldrich, #21604) at 50 µM to inhibit protein S-palmitoylation; 2-hydroxymyristic acid (2-HM, Cayman Chemical (Ann Arbor, MI, USA), #2507-55-3) at 100 µM to inhibit N-myristoylation; the farnesyltransferase inhibitor B581 (FTase Inhibitor I, Cayman Chemical, #22755) at 100 µM; GGTI-298 (geranylgeranyltransferase I inhibitor, Sigma-Aldrich, #G5169) at 10 µM; and NE10790 (geranylgeranyltransferase II inhibitor, MedChemExpress (Monmouth Junction, NJ, USA), #HY-16011) at 200 µM.

### 4.9. Statistical Analysis

Data were analyzed using Excel (Microsoft), IGOR Pro 6.0 (WaveMetrics), and GraphPad Prism 8.0 (GraphPad Software, San Diego, CA, USA). Values are reported as mean ± SEM, with n denoting the number of cells, fields, or independent cultures as indicated in the figure legends. Unless stated otherwise, each experiment was performed with at least three independent biological replicates. For statistical analysis: two-group comparisons utilized two-tailed unpaired Student’s *t*-tests. Comparisons among more than two groups employed one-way ANOVA followed by Dunnett’s post hoc multiple-comparisons test, as specified in the legends. Differences were considered significant at *, *p* < 0.05; **, *p* < 0.01; and ***, *p* < 0.001.

## 5. Conclusions

This study defines a multi-step trafficking pathway for the depalmitoylase ABHD17A, where site-specific palmitoylation within a conserved N-terminal cysteine cluster serves as a “code” that dictates its intracellular distribution and function. Palmitoylation of the middle region (C14, C15) is the key determinant for plasma membrane (PM) targeting and activity toward membrane-bound substrates like H-Ras, while the front (C10, C11) and rear (C18) positions modulate endosomal routing to influence PM delivery. These findings support the concept that multisite palmitoylation can encode distinct trafficking outcomes, and align with emerging perspectives on the roles of Golgi electrostatics and thioesterases in regulating the palmitoylation cycle.

## Data Availability

The original contributions presented in this study are included in the article/Supplementary Materials. Further inquiries can be directed to the corresponding author.

## Supplementary Materials

The following supporting information can be downloaded at https://www.mdpi.com/article/10.3390/ijms262010190/s1.

## Abbreviations

The following abbreviations are used in this manuscript:

ABHD17	α/β hydrolase domain-containing protein 17
2-BP	2-bromopalmitate
Acyl-RAC	acyl resin assisted capture
Acyl-PEG	acyl-polyethylene glycol
APT	acyl protein thioesterase
PPT1	palmitoyl protein thioesterase 1
PM	plasma membrane
zDHHC-PAT	zinc finger and DHHC motif containing palmitoyl acyltransferase
PI4KIIIα	phosphatidylinositol 4-kinase IIIα
PH-PLCδ1	pleckstrin homology domain of phospholipase C-δ1
SNAP25	synaptosome associated protein 25
