# Supplementary material for: Palmitoylation Code and Endosomal Sorting Regulate ABHD17A Plasma Membrane Targeting and Activity
Source: Int J Mol Sci. 2025 Oct 20;26(20):10190. doi: 10.3390/ijms262010190 (PMC12562545; doi:10.3390/ijms262010190)
Supplement: Supplementary file 1 [file ijms-26-10190-s001.zip › ijms-3910905-supplementary.pdf]

Supporting Information

Palmitoylation code and endosomal sorting regulate ABHD17A plasma membrane targeting and activity

Byeol-I Kim †, Jun-Hee Yeon † and Byung-Chang Suh*

Department of Brain Sciences, Daegu Gyeongbuk Institute of Science and Technology (DGIST), Daegu 42988, Republic of Korea; starkim@dgist.ac.kr (B.-I.K.); jhyeon@dgist.ac.kr (J.-H.Y.)

* Correspondence: bcsuh@dgist.ac.kr; Tel.: +82-53-785-6123

† These authors contributed equally to this work.

13 Supplementary Figures

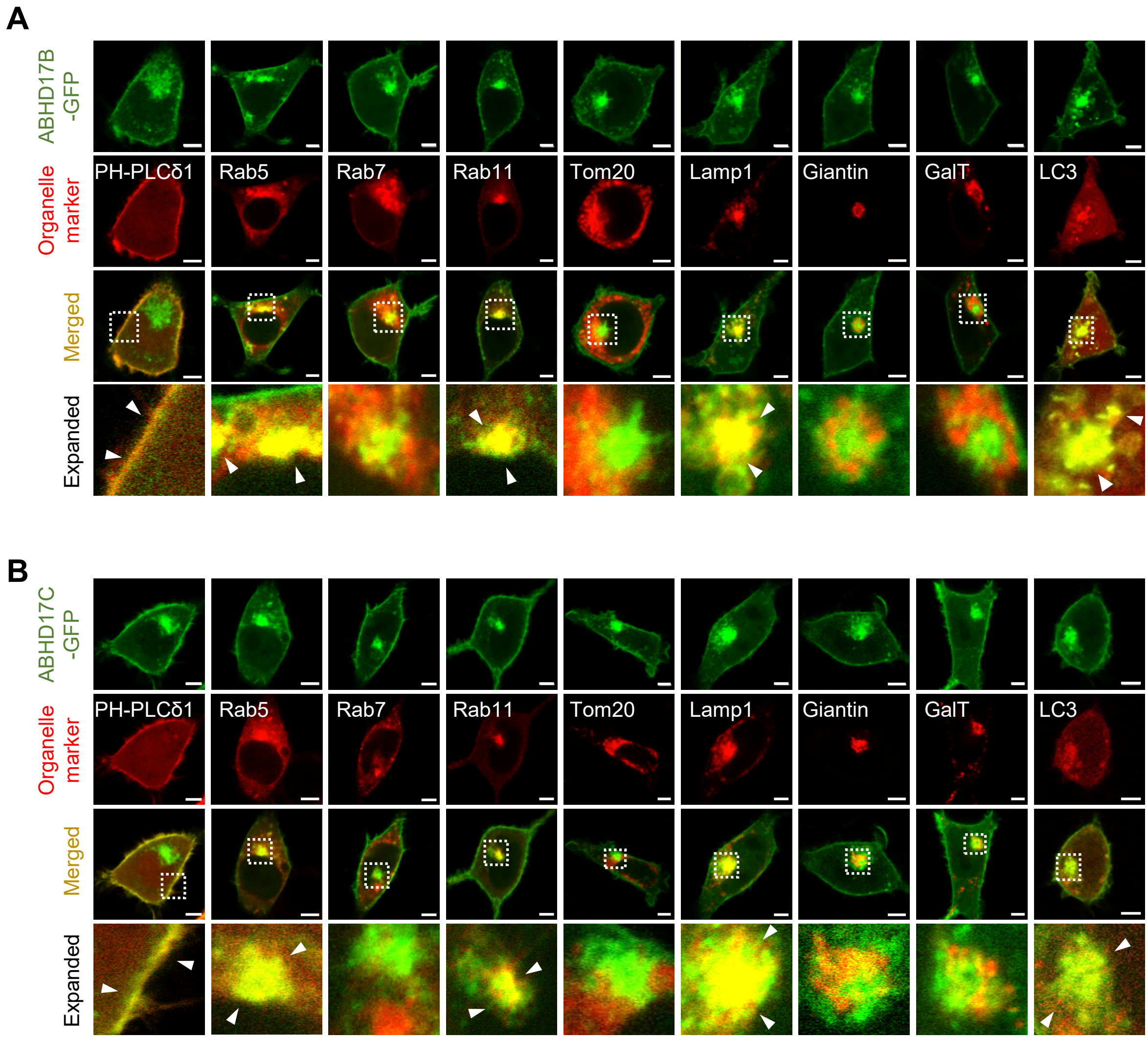

Figure S1. Subcellular localization of ABHD17B and ABHD17C in HEK293T cells. (A,B) Representative confocal images of cells expressing ABHD17B-GFP (A) or ABHD17C-GFP (B) together with RFP fused organelle markers : PH-PLCδ1 (PM), Rab5 (early endosome), Rab7 (late endosome), Rab11 (recycling endosome), Tom20 (mitochondria), Lamp1 (lysosome), Giantin (medial- and cis-Golgi), GalT (trans-Golgi network), and LC3 (autophagosome). Dashed boxes in the merged panels are enlarged in the bottom row, white arrowheads indicate co-localization between GFP and RFP. Scale bars, 5 μm.

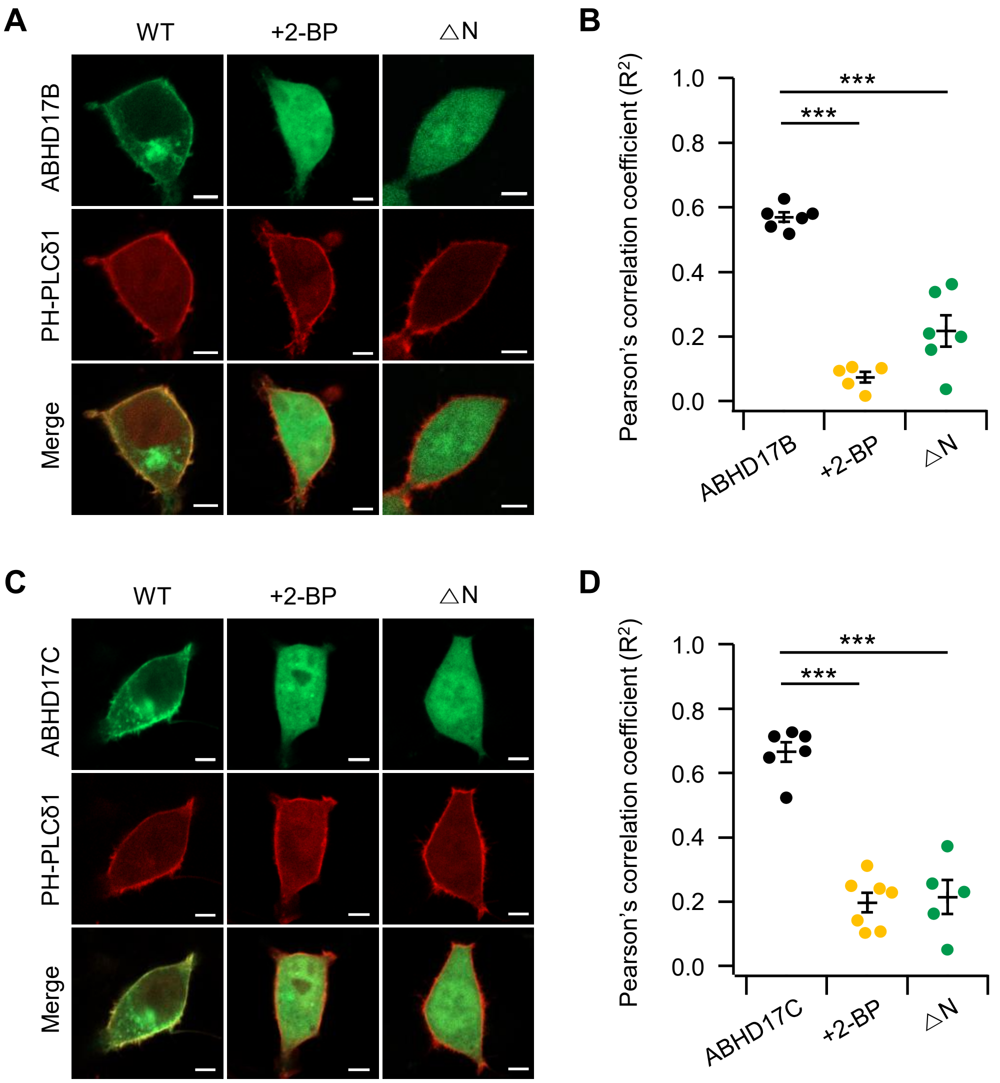

Figure S2. N-terminal palmitoylation is required for PM localization of ABHD17B and ABHD17C. Representative confocal images of HEK293T cells expressing ABHD17B-GFP (A) or ABHD17C-GFP (C) together with the PM marker PH-PLCδ1. Conditions shown are wild type (WT), 2-BP treatment (50 µM, 12-24 h), and △N (N-terminal deletion). Scale bars: 5 µm. (B,D) Quantification of PM association for ABHD17B (B) and ABHD17C (D) was performed on whole-cell ROIs using Pearson's correlation (R^2) between GFP and PH-PLCδ1. Each symbol represents one cell. Data are mean ± SEM. ***, $P < 0.001$, with one-way ANOVA followed by Dunnett's post-hoc test.

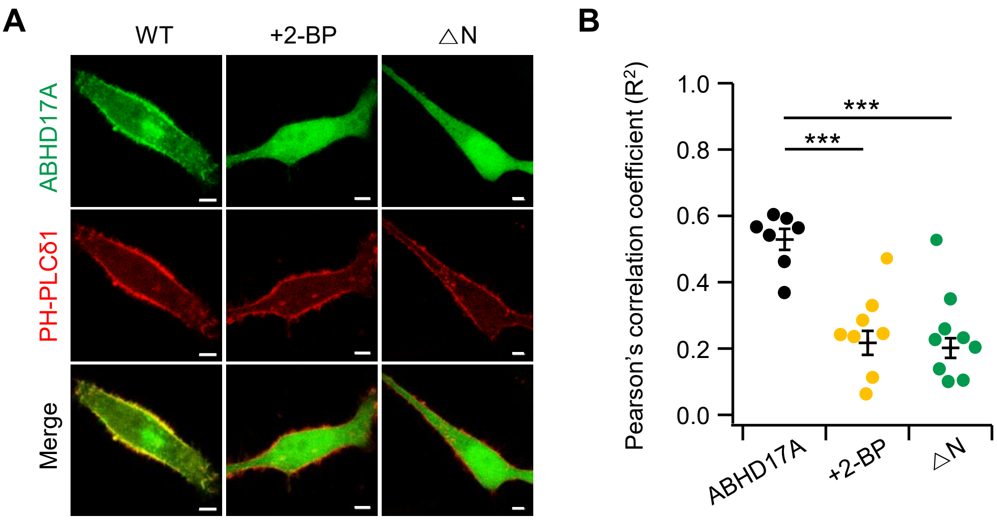

Figure S3. N-terminal palmitoylation is required for PM localization of ABHD17A in HeLa cells. (A) Representative confocal images of HeLa cells expressing ABHD17A-GFP together with the PM marker PH-PLCδ1. Conditions shown are wild type (WT), 2-BP treatment (50 µM, 12-24 h), and △N (N-terminal deletion). Scale bars: 5 µm. (B) Quantification of colocalization between ABHD17A signals and PH-PLCδ1 was performed on whole-cell ROIs using Pearson's correlation coefficient (R^2) values. Each symbol represents one cell. Data are mean ± SEM. ***, $P < 0.001$, with one-way ANOVA followed by Dunnett's post-hoc test.

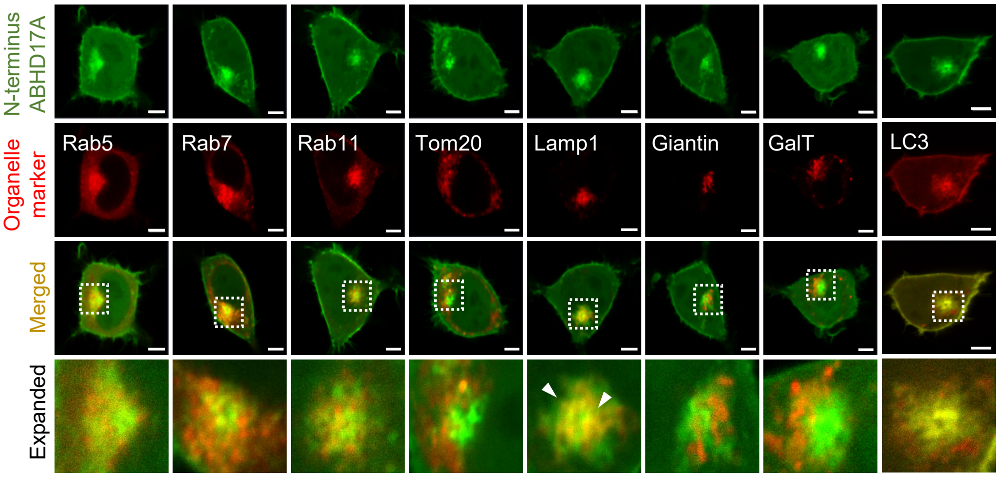

Figure S4. Subcellular localization of the isolated N-terminus of ABHD17A. Representative confocal images of HEK293T cells expressing ABHD17 N-terminus (N1-19)-GFP together with organelle markers: Rab5 (early endosome), Rab7 (late endosome), Rab11 (recycling endosome), Tom20 (mitochondria), Lamp1 (lysosome), Giantin (medial- and cis-Golgi), GalT (trans-Golgi network), and LC3 (autophagosome). Dashed boxes in the merged panels are enlarged in the bottom row, white arrowheads indicate co-localization between GFP and RFP. Scale bars, 5 μm.

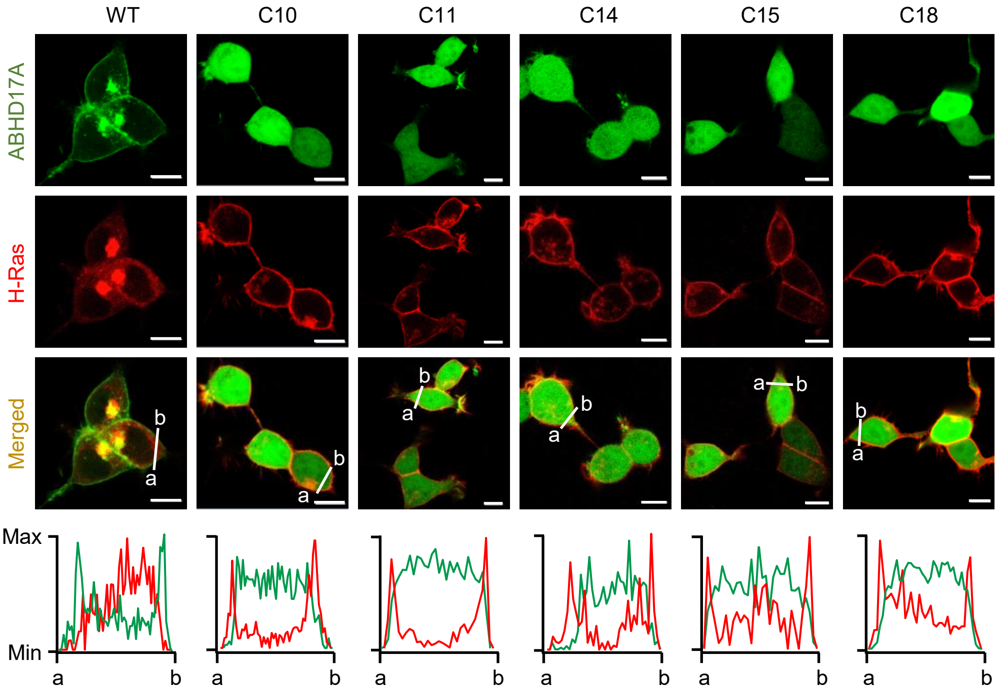

Figure S5. Single-cysteine variants of ABHD17A insufficient to depalmitoylate H-Ras. Representative confocal images of HEK293T cells expressing ABHD17A-GFP (WT or single-cysteine variants) and mCherry-H-Ras. Each image shows an independent multicell field acquired under the same imaging settings as Figure 2D from replicate experiments. Scale bars: 10 μm. Line-scan plots along the indicated a-b lines display normalized fluorescence intensity profiles of ABHD17A (green) and H-Ras (red).

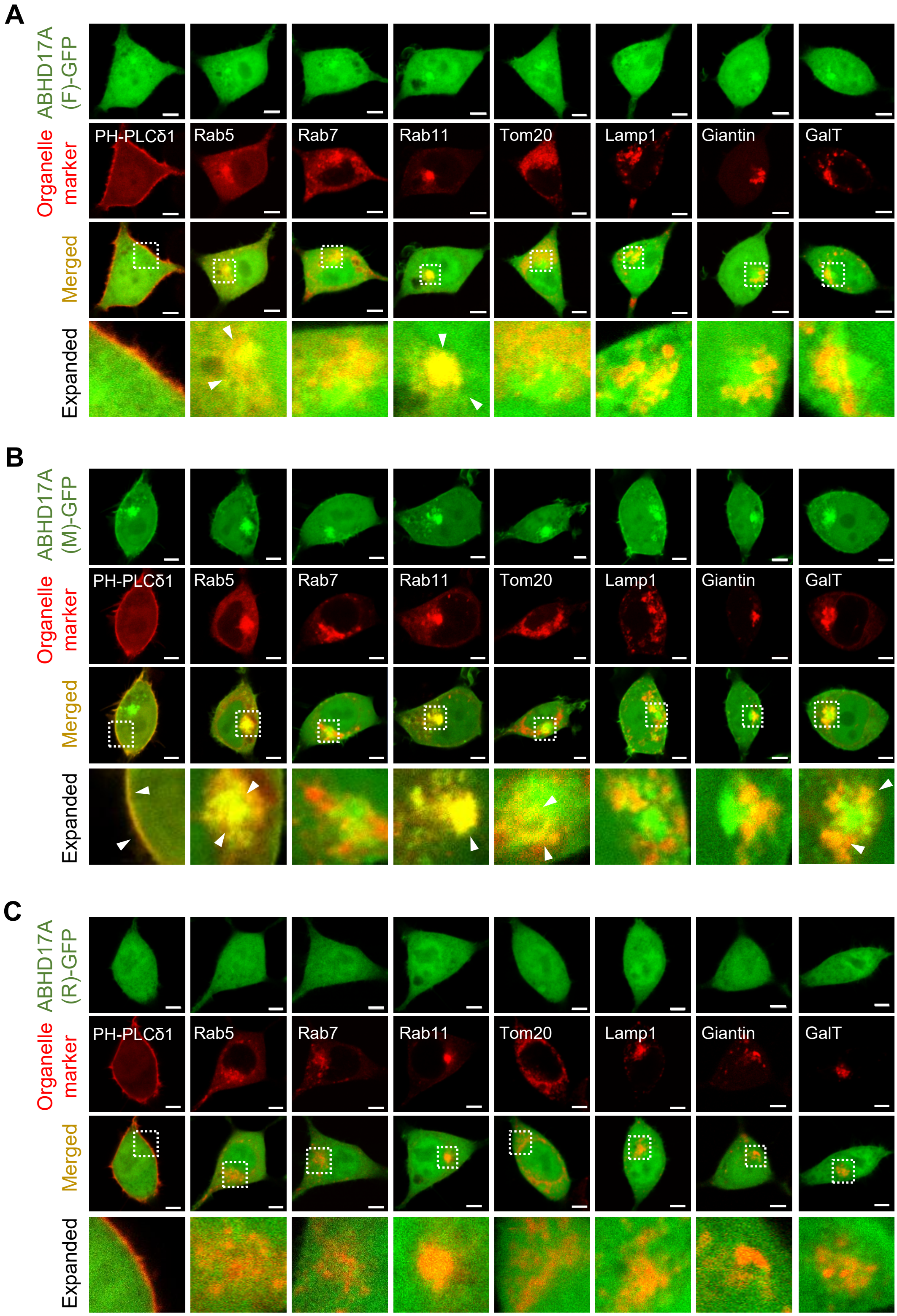

Figure S6. Subcellular localization of single-region ABHD17A constructs (F, M, R). (A-C) Representative confocal images of HEK293T cells expressing ABHD17 variants that retain only one region of N-terminal cysteines and fused to GFP. Cells were co-imaged with organelle markers PH-PLCδ1 (PM), Rab5 (early endosome), Rab7 (late endosome), Rab11 (recycling endosome), Tom20 (mitochondria), Lamp1 (lysosome), Giantin (medial- and cis-Golgi), and GalT (trans-Golgi network). Dashed boxes in the merged panels are enlarged in the bottom row, white arrowheads indicate co-localization between GFP and RFP. Scale bars, 5 µm.

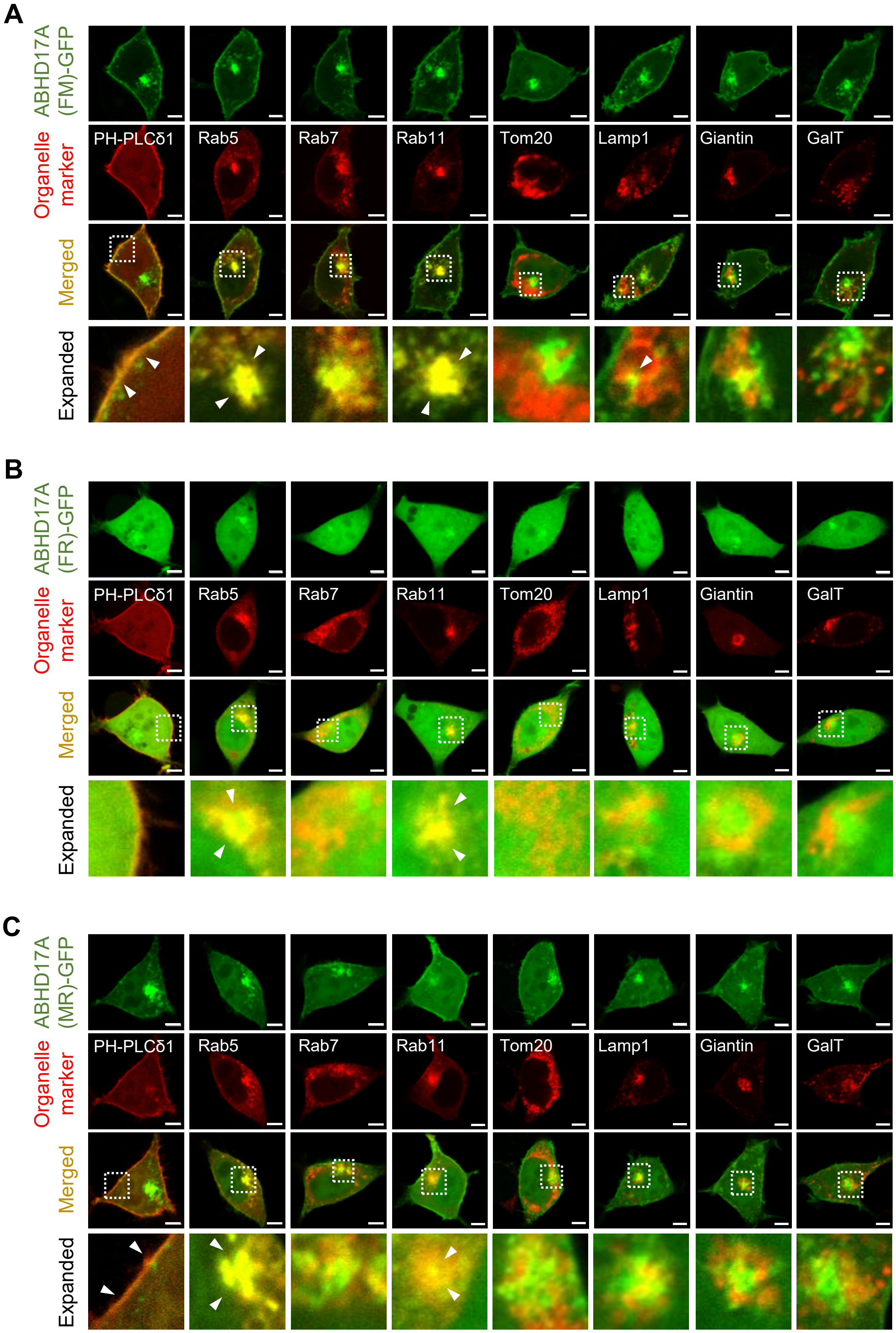

Figure S7. Subcellular localization of dual-region ABHD17A constructs (FM, FR, MR). (A-C) Representative confocal images of HEK293T cells expressing ABHD17-GFP that retain two regions within the N-terminal cluster. The same organelle markers as in Figure S6 were used. Dashed boxes in the merged panels are enlarged in the bottom row, white arrowheads indicate co-localization between GFP and RFP. Scale bars: 5 μm.

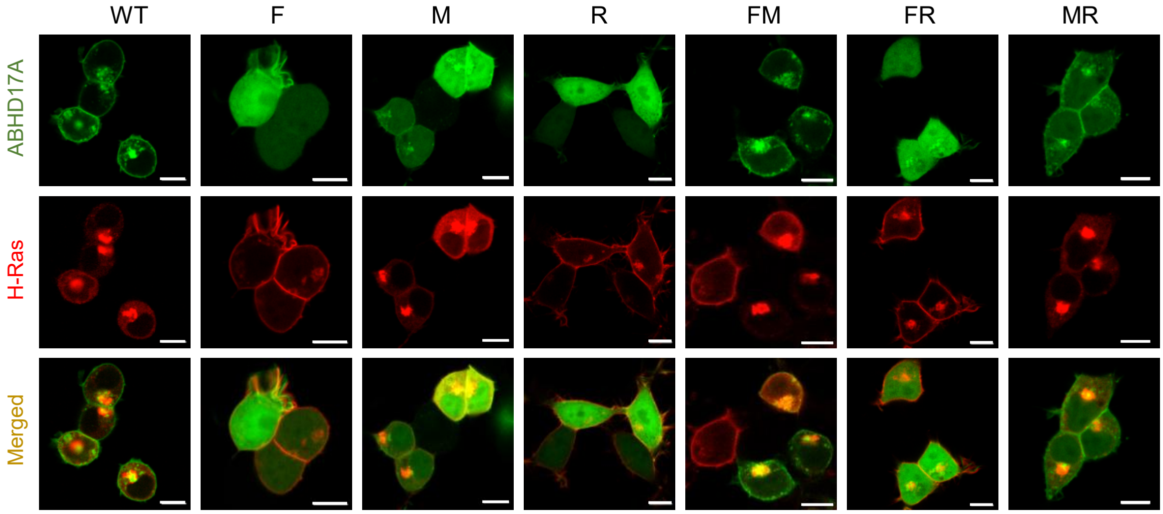

Figure S8. Multi-cell images corresponding to Figure 3F. Representative confocal images of HEK293T cells co-expressing ABHD17A-GFP (WT, F, M, R, FM, FR, MR) and mCherry-H-Ras. Each image shows an independent multicell field acquired under the same imaging settings as Figure 3F from replicate experiments. Scale bars: 10 μm.

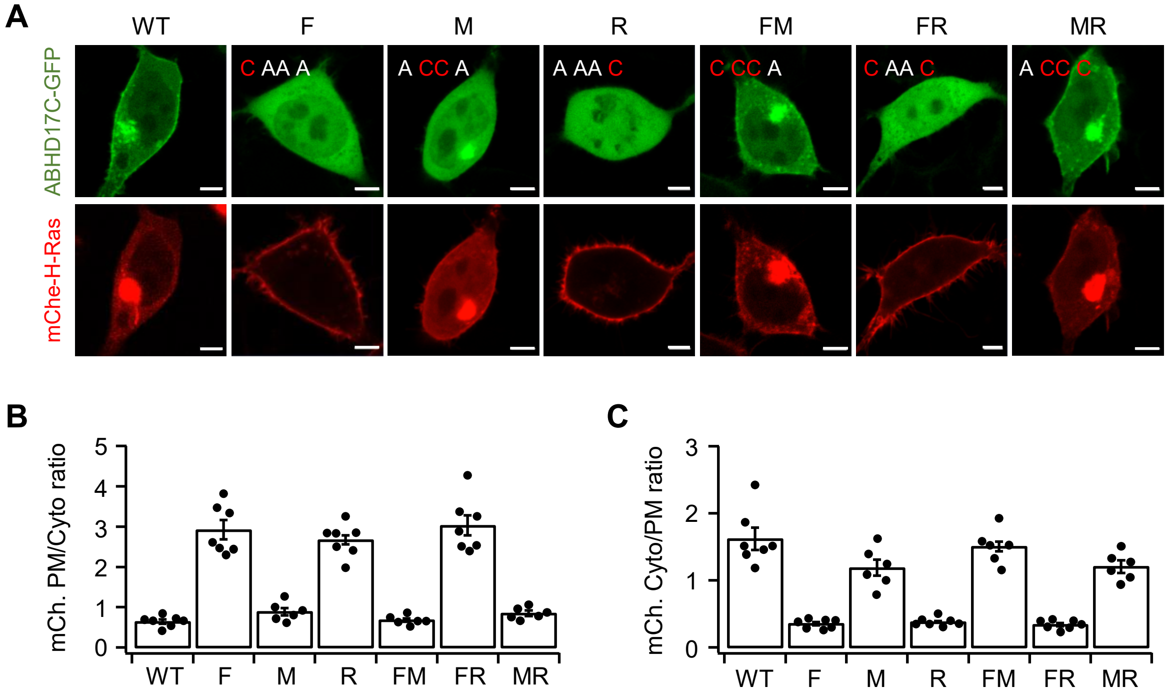

Figure S9. ABHD17C requires the middle region code to promote H-Ras depalmitoylation. (A) Representative confocal images of HEK293T cells co-expressing mCherry-H-Ras and ABHD17C-GFP constructs: WT, single-region F (retains C10/C11), M (retains C14/C15), R (retains C18), and dual-region FM, FR, MR (as defined in Fig. 3). Scale bars: 5 µm. (B,C) Quantification of mCherry-H-Ras distribution measured as the mCherry fluorescence ration at the PM versus cytosol (PM/Cyto, B) and the reciprocal Cyto/PM ration (C).

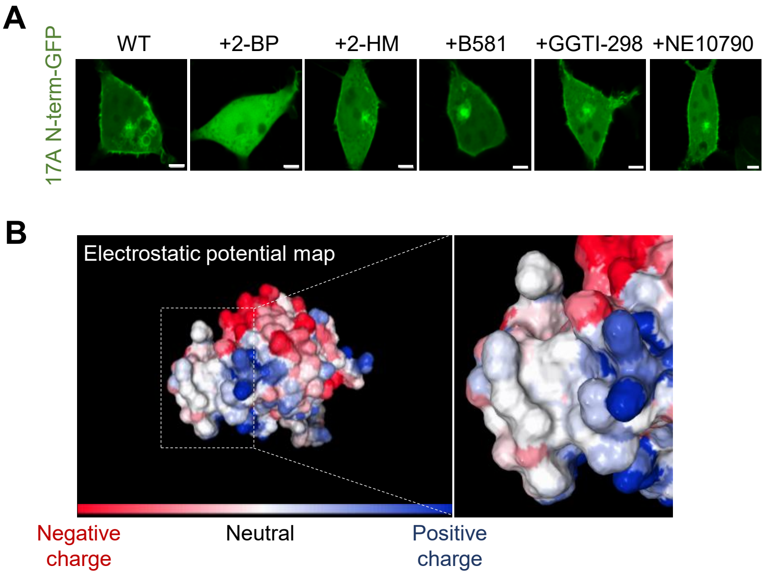

Figure S10. Inhibitor sensitivity and electrostatic surface of the ABHD17A N-terminus. (A) Representative confocal images of HEK293T cells expressing the ABHD17A N-terminus-GFP (N1-19) after treatment for 12-24 h with 2-BP, 2-HM, B581, GGTI-298, or NE10790. Scale bars: 5 µm. (B) Electrostatic potential map of ABHD17A viewed from the N-terminal face with an enlarged view of the N-terminal segment at right. Colors denote negative (red), neutral (white), and positive (blue) potential, indicating an approximately neutral surface near the N-terminus.

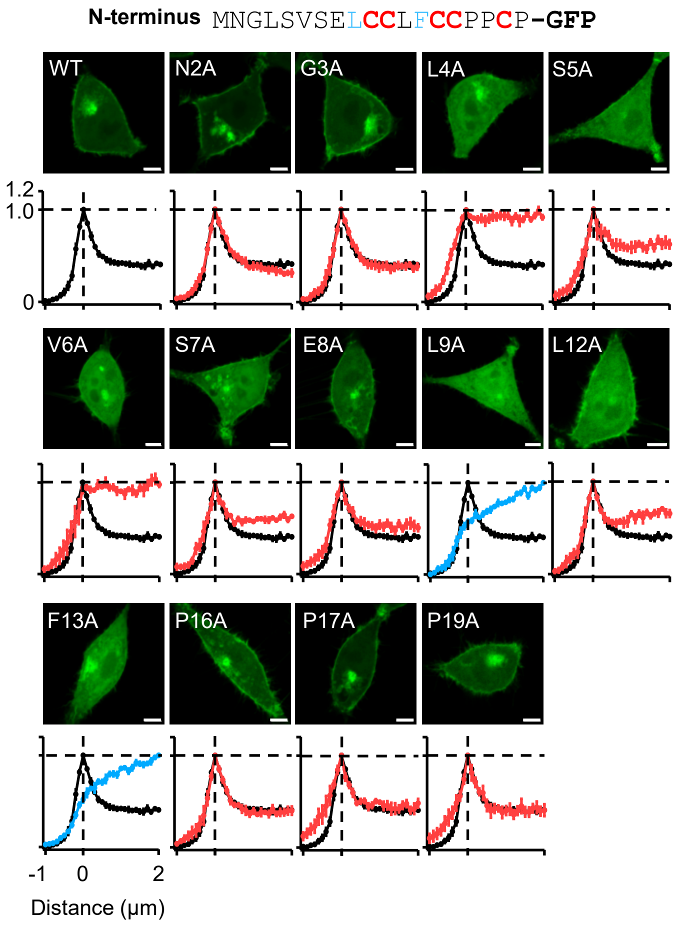

Figure S11. Alanine scan of the ABHD17A N-terminus identifies residues that support PM association. Top, ABHD17A N-terminal sequence (N-19) with palmitoylated cysteines in red. Representative confocal images of HEK293T cells expressing N-terminus-GFP variants in which each non-cysteine residue was individually substituted with alanine (N2A, G3A, L4A, S5A, V6A, S7A, E8A, L9A, L12A, F13A, P16A, P17A, P18A). Scale bars: 5 µm. Traces below each image show normalized line-scan fluorescence across the indicated line. Black, WT reference; red, mutant; blue, L9A and F13A, which most strongly reduce PM association.

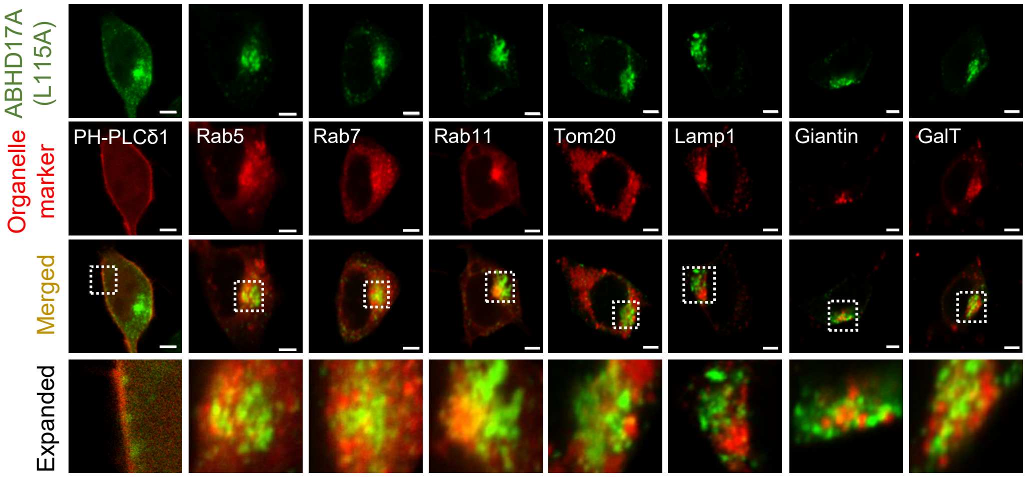

Figure S12. Subcellular localization of the ABHD17A L115A mutant. Representative confocal images of HEK293T cells expressing ABHD17A-L115A-GFP together with organelle markers. The same organelle markers as in Figure S1 were used. Dashed boxes in the merged panels are enlarged in the bottom row. Scale bars: 5 μm.

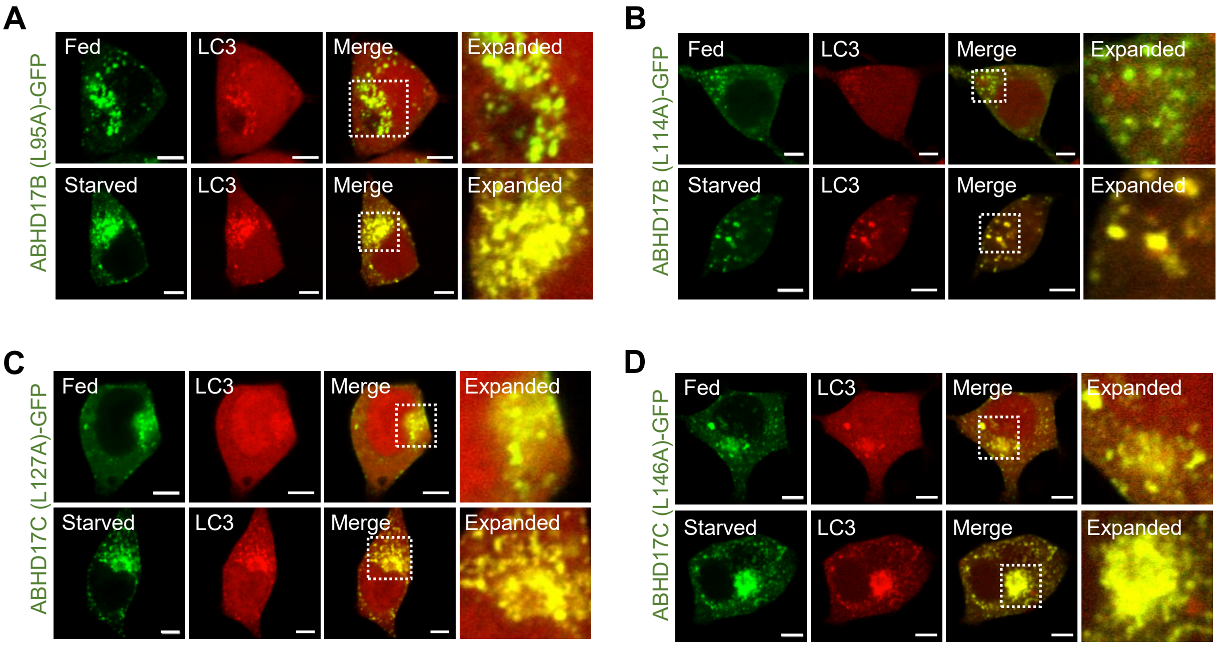

Figure S13. Endosomal-motif mutants of ABHD17B and ABHD17C accumulate in autophagosomes upon starvation. Representative confocal images of HEK293T cells expressing GFP-tagged ABHD17B or ABHD17C mutants together with the autophagosome marker LC3. Cells were imaged either in growth medium (Fed) or after serum-starvation for 2-4 h in serum free DMEM (Starved). (A) ABHD17B(L95A)-GFP. (B) ABHD17B(L114A)-GFP. (C) ABHD17C(L127A)-GFP. (D) ABHD17C(L146A)-GFP. Dashed boxes mark regions enlarged at right. Scale bars: 5 µm.
